# Fifth Annual Conference at Newcastle-On-Tyne, June 18 to 20, 1914

**Published:** 1914-06-27

**Authors:** 


### THE BRITISH HOSPITALS ASSOCIATION.

### FIFTH ANNUAL CONFERENCE AT NEWCASTLE-ON-TYNE, JUNE 18 to 20, 1914.

The fifth annual Conference of the British Hospitals Association took place at Newcastle-on-Tyne on Thursday, June 18, and Friday, June 19, 1914, by kind permission of the Governors, in the library of the Royal Victoria Infirmary, Queen Victoria Road (of which we published an illustration last week), under the presidency at the outset of the Right Honourable the Lord Mayor of Newcastle-on-Tyne, Councillor Johnstone Wallace, J.P. There was a large attendance.

Sir George Hare Philipson, M.D., D.C.L., J.P., Chairman of the Royal Victoria Infirmary, Newcastle, the Deputy-Chairman of the Conference, said he had attended there as a member of the British Hospitals Association and also in his position as the Chairman of the house committee of the Royal Victoria Infirmary, Newcastle, to offer to the members of the British Hospitals Association a hearty and cordial welcome on the first occasion that the British Hospitals Association had arranged for their annual meeting to take place in that city, and to wish every success to the Conference. At that moment he was busily occupied with examinations for degrees in medicine of the University of Durham, and he had come from the wards in that infirmary to receive them and also to express the kind feelings of the house committee towards them. Having performed that duty he should ask the permission of the Lord Mayor to leave the gathering in order to resume his examination duties, but he was looking forward with pleasure to meeting the visitors at luncheon later in the day when they would be the guests of the house committee of that institution.

